# Implementation of a real‐time neutron monitor system for use in routine quality assurance of an accelerator‐based neutron system for clinical boron neutron capture therapy

**DOI:** 10.1002/acm2.70190

**Published:** 2025-07-25

**Authors:** Naonori Hu, Taiki Nakamura, Ryusuke Kataura, Keita Suga, Tetsuya Mukawa, Kazuhiko Akita, Ryo Kakino, Akinori Sasaki, Mai Nojiri, Nishiki Matsubayashi, Takushi Takata, Hiroki Tanaka, Keiji Nihei, Koji Ono

**Affiliations:** ^1^ Kansai BNCT Medical Center Osaka Medical and Pharmaceutical University Osaka Japan; ^2^ Institute for Integrated Radiation and Nuclear Science Kyoto University Osaka Japan; ^3^ Sumitomo Heavy Industries, Ltd. Tokyo Japan; ^4^ Graduate School of Engineering Kyoto University Kyoto Japan; ^5^ Department of Radiation Oncology Osaka Medical and Pharmaceutical University Hospital Osaka Japan

**Keywords:** accelerator neutron source, BNCT, Monte Carlo simulation, QA, Real‐time neutron monitor

## Abstract

**Background:**

Currently, the metal foil activation method is routinely used to measure the neutron output of an accelerator‐based neutron source designed for clinical Boron neutron capture therapy (BNCT). Although this method is well established and has been primarily utilized since the nuclear reactor BNCT era, the process is labour‐intensive and not well‐suited for a busy hospital environment performing routine patient treatment. A replacement neutron detector system that is simple to use and can measure the neutron output in real‐time is necessary.

**Purpose:**

Investigation and implementation of an Eu doped LiCaAlF_6_ scintillator detector for use in routine quality assurance tests of an accelerator‐based neutron source designed for clinical BNCT.

**Methods:**

The response of the scintillator detector was evaluated using the NeuCure BNCT system installed at the Kansai BNCT Medical Center. The measurement repeatability, neutron fluence linearity, and neutron flux dependency of the detector system were evaluated. The beam central axis and off‐axis thermal neutron distribution inside a water phantom were measured and compared with the Monte Carlo treatment planning system (TPS).

**Results:**

The scintillator detector system showed high measurement repeatability with a coefficient of variation of less than 0.4%. The detector system showed linear response up to a proton charge of 3.6 C, and the response was stable between a proton current of 0.1 and 1 mA. Both the central axis and off‐axis thermal neutron flux inside a water phantom matched closely with both the metal foil activation method and the Monte Carlo simulation results. The time it took to perform a routine quality assurance test was drastically reduced from 1.5 h down to a few minutes.

**Conclusion:**

Implementation of this detector system in the clinic would significantly reduce the time required for routine QA, acceptance, and commissioning, and be a stepping stone to assist expansion of accelerator‐based BNCT systems worldwide.

## 1 INTRODUCTION

Boron neutron capture therapy (BNCT) is a type of cancer treatment whereby the cancer cells are targeted at the cellular level. The nuclear reaction between a low‐energy neutron (thermal neutron) and a boron‐10 atom produces high linear energy transfer particles (alpha and ^7^Li), which deposit their energy over a short range.^1^ This ensures a localized dose delivered to the tumor while minimizing the dose delivered to the surrounding healthy tissue.

In recent years, the development of accelerators designed for performing clinical neutron capture therapy has rapidly increased. The world's first commercially available accelerator‐based neutron system (NeuCure) was developed by Sumitomo Heavy Industries, Ltd. and was installed at the Southern Tohoku General Hospital^2^ in 2015 and at Osaka Medical and Pharmaceutical University, Kansai BNCT Medical Center in 2018,^3^ both in Japan. This system, in conjunction with the boron drug Steboronine, developed by Stella Pharma corporation, received approval in June 2020 by the Ministry of Health, Labour and Welfare in Japan for the treatment of locally unresectable recurrent or unresectable advanced head and neck cancer.^4^ Various other countries are developing and installing an accelerator system for BNCT.^5^ With the increase in the number of facilities that are planning on performing clinical BNCT, the development of a fast and simple to use real‐time detector system for routine quality control of neutron output measurement is highly sought out.

In the past, ^3^He gas counters were used to measure thermal neutrons due to their high thermal neutron cross section and low gamma ray sensitivity.^6^ However, due to the declining resources of ^3^He gas from the increase in the supply of neutron detectors for security reasons, a replacement detector system is necessary. Scintillator neutron detectors with optical fiber have been developed in the past several years for BNCT applications.^7^ The scintillator for thermal neutron detection should consist of light elements to reduce sensitivity for gamma rays, high light yield, uniform light collection, fast decay time, and high transmittance. Eu doped LiCaAlF_6_ is useful for measuring thermal neutrons in a mixed neutron‐gamma ray environment. This detector utilizes the ^6^Li(n,t)α reaction induced by thermal neutrons and the reaction products (alpha and triton) excite the scintillator. These reaction products travel a short range (few tens of micrometers), while electrons (which are produced from gamma rays) have a relatively long range (few millimeters). So, by controlling the size of the scintillator crystal, the signal from the neutron reaction events will deposit their entire energy inside the scintillator, while the gamma ray events escape to outside the scintillator, which allows discrimination between neutron and gamma ray events. Few authors have used this type of scintillator for measurement in a low intensity neutron irradiation field by adopting an enriched ^6^Li (95%) crystal.^8^, ^9^ However, an enriched ^6^Li crystal is expensive and not ideal for mass production for commercial purposes. This paper investigates the suitability of a scintillator detector using natural Li as a routine detector for quality assurance purposes of an accelerator‐based neutron source designed for clinical BNCT.

## 2 METHOD

**FIGURE 1:**
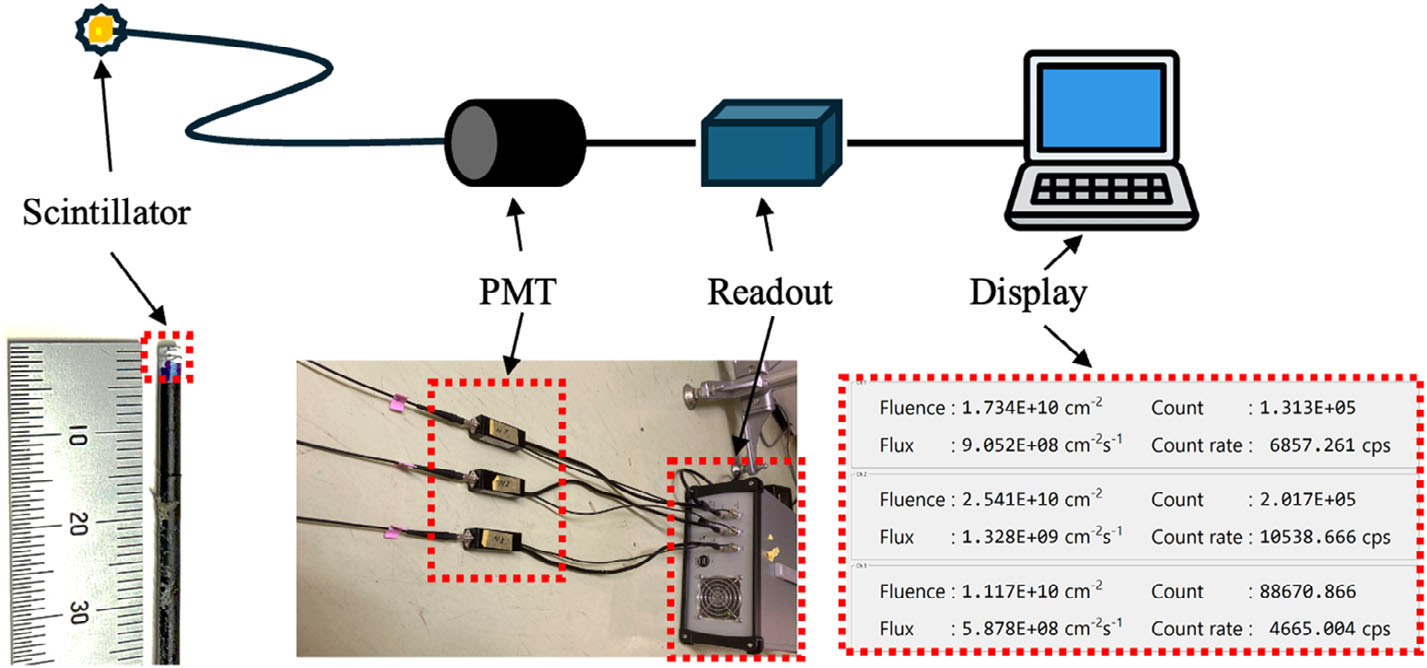
Diagram and image of the scintillator detector system.

A real‐time neutron fluence monitor developed by Sumitomo Heavy Industries was used for this study. This system consists of three units, an Eu:LiCaAlF_6_ scintillator detector (developed by C&A corporation, model number FD‐H(3‐2)Q0610), photo multiplier tube (developed by Hamamatsu Photonics K.K, model number H10722‐110), readout module system, and a software displaying the neutron fluence, flux, count, and count rate in real‐time. An image of the real‐time neutron fluence monitor system is shown in Figure 1. The scintillator detector specification is summarized in Table 1. Three scintillator detectors were used in this study. The scintillator detectors were calibrated using the standard thermal neutron field at the National Institute of Advanced Industrial Science and Technology (AIST). The conversion from raw detector signal to thermal neutron fluence was performed by calibrating it against the gold foil activation method.

**TABLE 1:** Specifications of the scintillator detector used in this study.

	Model FD‐H(3‐2)Q0610
Scintillator dimensions	0.3 × 0.3 × 0.2 mm^3^
Li‐6 abundance	Natural
Density	2.98 g/cm^3^
Eu concentration	< 0.1 %
Optical fiber core diameter	0.6 mm
Optical fiber outer diameter	< 3 mm
Connector	SMA

**FIGURE 2:**
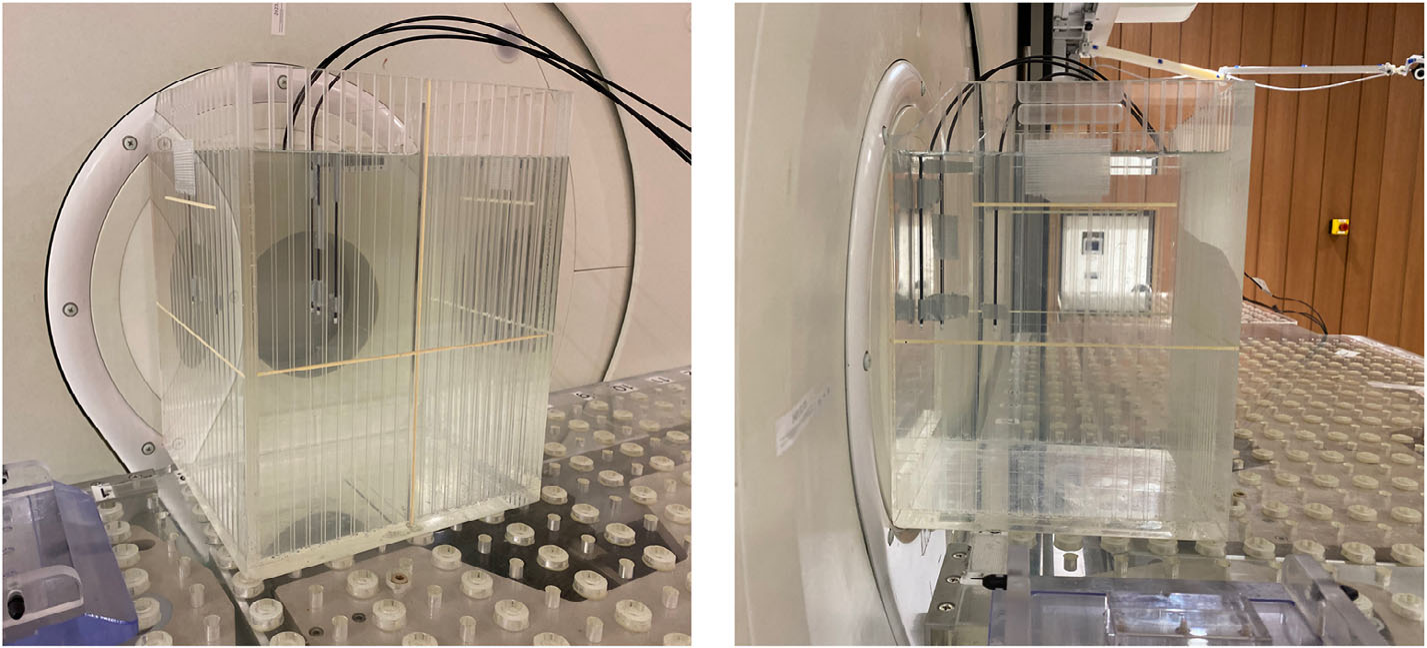
Image of the experimental setup. The scintillators are placed vertically into the water phantom.

Experimental measurements were performed using the NeuCure® BNCT system installed at the Kansai BNCT Medical Center. A reference collimator size of 12 cm in diameter was used. The scintillator detector was placed inside an acrylic walled phantom filled with water (20 cm×20 cm×20 cm), shown in Figure 2. The following tests were performed.

### 2.1 Repeatability

The repeatability measurement of the scintillator was evaluated by placing the detector at a depth of 4.5, 20, and 60 mm inside a water phantom. The depth of 20 and 60 mm correspond to the reference depths for routine quality assurance of neutron output.^10^ A proton charge of 0.1 C was delivered, and the measurements were repeated three times. The raw detector count was divided by the delivered proton charge to obtain the detector counts per coulomb. The neutron fluence was calculated by multiplying the raw detector count with the calibration factor, explained above. The standard deviation (SD) and the coefficient of variation (CV) were calculated using the following expression:

$$SD=\sqrt{\frac{\sum {\left(x_{i}-\mu \right)}^{2}}{N}}$$

$$CV=\frac{SD}{\mu }$$

where *x_i_* is each value from the population size *N*, and *μ* is the population mean.

### 2.2 Neutron fluence response (linearity)

The neutron fluence response of the scintillator was evaluated by varying the proton charge and measuring the number of detected counts. Proton charges 0.1, 0.2, 0.3, 0.4, 0.6, 1.2, and 3.6 C were set and delivered to the scintillator placed at a depth of 4.5, 24.5, and 107 mm inside the water phantom along the beam central axis. Measurements were repeated twice for each proton charge.

### 2.3 Neutron flux response

The neutron flux response of the scintillator was evaluated by varying the proton current of the accelerator and measuring the number of detected counts. Proton currents 100, 200, 400, 600, 800, and 1000 µA were set, and a proton charge of 0.1 C was delivered to the scintillator placed at a depth of 20 mm inside the water phantom along the beam central axis. Measurements were repeated twice for each proton current.

### 2.4 2D profile measurement

The central axis depth profile and off‐axis profile were evaluated by placing the scintillator detector at various depths and locations inside the water phantom. A proton charge of 0.1 C was delivered for each measurement. The measurements were compared with the gold foil activation measurement. Furthermore, the results were compared with the treatment planning system (TPS), NeuCure dose engine, which uses the PHITS Monte Carlo code for neutron transport.^11^

## 3 RESULTS

### 3.1 Repeatability

The average number of counts per coulomb at each depth and the corresponding thermal neutron fluence is shown in Table 2. The CV was calculated to be less than 0.4% at all three depths.

**TABLE 2:** Results of the repeatability measurements performed with the scintillator detector as three different depths.

	Average		SD		CV (%)	
Depth (mm)	Counts/C	Fluence/C	Counts/C	Fluence/C	Counts/C	Fluence/C
4.5	7.01E+06	9.25E+11	2.41E+04	3.18E+09	0.34	0.34
20	1.09E+07	1.38E+12	3.02E+04	3.81E+09	0.28	0.28
60	4.80E+06	6.05E+11	1.38E+04	1.73E+09	0.29	0.29

### 3.2 Neutron fluence response (linearity)

**FIGURE 3:**
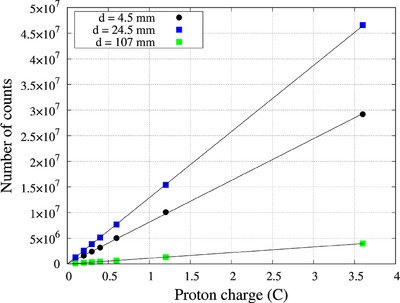
Dose response curve of the scintillator detector at three different depths.

The response in the scintillator detector with varying neutron fluence (i.e., varying the total proton charge delivered) at three different depths is shown in Figure 3. The detector showed a linear response between 0.1 and 3.6 C, with a high coefficient of determination of > 0.99 at all three depths.

### 3.3 Neutron flux response

**FIGURE 4:**
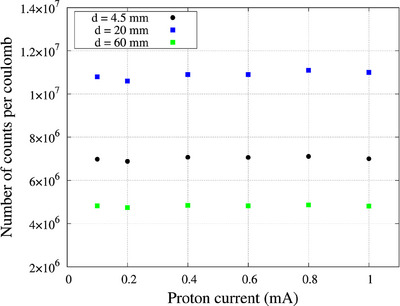
Dose rate response of the scintillator detector at three different depths.

The response in the scintillator detector with varying neutron flux (i.e., varying proton current) at three different depths is shown in Figure 4. The CV was calculated to be 1.2%, 1.3%, and 0.9% at the depths of 4.5, 20, and 60 mm, respectively.

### 3.4 2D profile measurement

**FIGURE 5:**
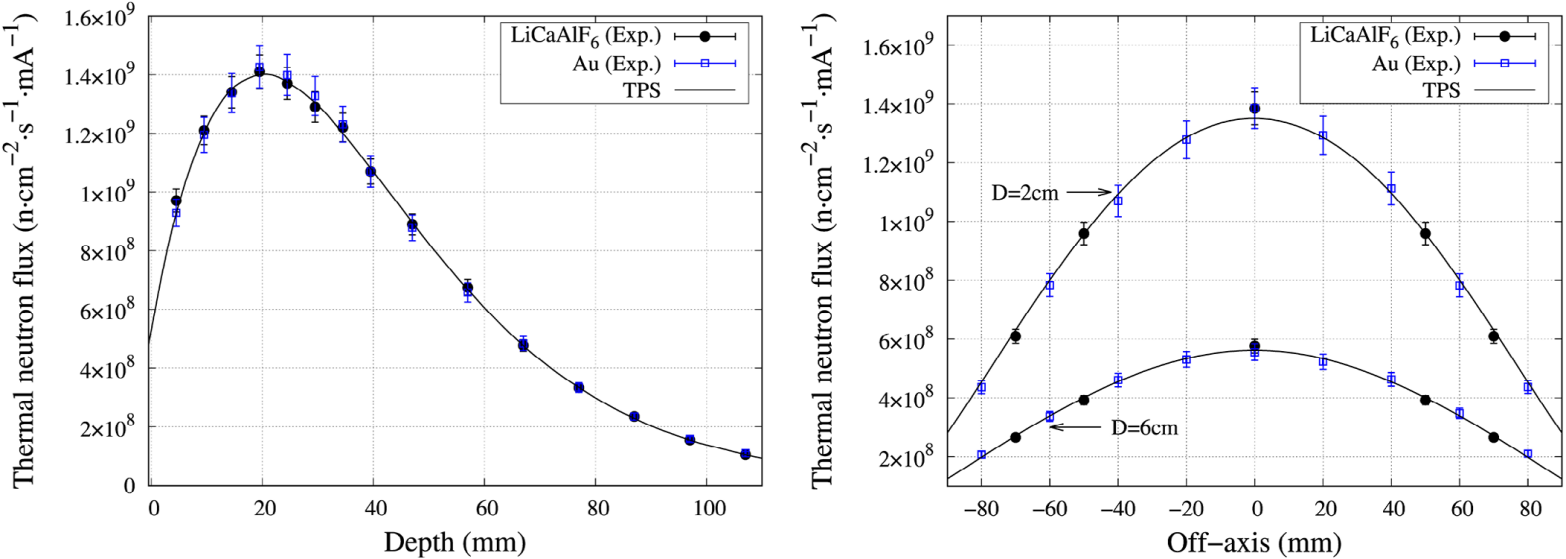
(Left) Central axis thermal neutron flux distribution curve measured with the scintillator detector and the gold foil activation method. (Right) Off‐axis thermal neutron flux distribution measured with the scintillator detector and the gold foil activation method at a depth of 2 and 6 cm in water.

The central axis and off‐axis thermal neutron flux distribution is shown in Figure 5. The thermal neutron flux measured with the scintillator closely resembled the gold foil activation measurements. Both measurements with the scintillator and the gold foil closely matched the thermal neutron flux calculated by the TPS.

## 4 DISCUSSION

The real‐time neutron fluence monitor developed by Sumitomo Heavy Industry was evaluated using the NeuCure BNCT system installed at the Kansai BNCT Medical Center. The detector was found to have a linear response to proton charge, up to 3.6 C, and independent to the proton current, between 0.1 and 1 mA. From a clinical perspective, the NeuCure BNCT system operates at a maximum proton current of 1 mA and the maximum proton charge delivered in a single irradiation is 3.6 C.^12^ Therefore, from a clinical point of view, this scintillator detector system is suitable to be used under a clinical setting. The central axis and off‐axis thermal neutron distribution inside a water phantom agreed well with Monte Carlo simulation results.

At the Kansai BNCT Medical Center, the time required to perform weekly dosimetry QA takes approximately one and a half hours. Two irradiations are performed (one with bare gold and another with bare gold covered in cadmium), 5 and 10 min, respectively. Following irradiation, the activation of the gold wire is measured using a germanium detector. This process takes approximately 5–15 min per gold wire, depending on the depth at which the gold wire was placed (measurements are performed at three different depths: 0, 2, and 6 cm). A study performed by Suzuki et al., showed the optimum proton charge and measurement time for gold activation method was found to be approximately 7 C and 900 s for bare gold and 54 C and 900 s at 0–2.2 cm depth and 3600s at deeper depths with cadmium cover, for an accelerator‐based BNCT system utilizing a lithium target.^13^ Given the neutron intensity varies between different accelerators, by normalizing the thermal neutron intensity, the irradiation time becomes approximately 2 min for bare gold and 15 min for the cadmium covered gold, followed by 900s measurement using the germanium detector for each bare gold wire and cadmium covered gold wire between 0–2.2 cm depth and 3600 s for each cadmium covered gold wire greater than a depth of 2.2 cm, for the NeuCure BNCT system (with the germanium detection efficiency equal to approximately 5%). This would result in a total time of approximately two and a half hours to perform weekly QA with a total uncertainty of 4.5 %.

Using the real‐time neutron fluence monitor system, the proton charge necessary to keep the measurement reproducibility uncertainty below 0.5 % was 0.1 C, which equates to 100 s when the accelerator is operated at 1 mA. The readout system is capable of processing three scintillator detectors simultaneously, so all three measurements (near surface, 2, and 6 cm) were completed in 100 s. The readout system displays the thermal neutron fluence instantaneously, so no offline data processing was required. Furthermore, repeating the measurement is simple, whereas for the gold activation method, a new gold wire will need to be prepared and analyzed. This scintillator detector will significantly reduce the time it takes to perform routine QA. The total experimental uncertainty of the real‐time neutron fluence monitor system (calibration of the detector (3%), experimental setup (1%), neutron fluence dependency (1%), and neutron flux dependency (1.3%)) was estimated to be approximately ± 4%, which is comparable to the gold foil activation method. As mentioned earlier, BNCT irradiation field consists of both neutrons and gamma rays, and it is important to perform routine quality control tests for both components. This detector system can be used for routine neutron output measurement and an optically simulated luminescent dosimeter (OSLD) has been showed to be effective for routine gamma ray output measurement of an accelerator‐based neutron source.^14^ The combination of these two detector systems will provide a complete dosimetry system for routine quality assurance of an accelerator‐based neutron source designed for BNCT.

Future work will involve investigating the use of this new scintillator detector in combination with a 3D scanning water phantom to further optimise the QA and commissioning procedure of an accelerator‐based neutron source.

## 5 CONCLUSION

The Eu:LiCaAlF_6_ scintillator detector proved to be useful in measuring the thermal neutron fluence of an accelerator‐based neutron source designed for clinical BNCT. The implementation of this detector system for routine dosimetry QA will significantly improve the workflow efficiency and expedite the acceptance and commissioning process.

## AUTHOR CONTRIBUTIONS

Conceptualization and methodology, Naonori Hu and Taiki Nakamura; Data analysis and process, Ryusuke Kataura, Keita Suga, Tetsuya Mukawa, Akinori Sasaki, and Mai Nojiri; Resources, Kazuhiko Akita, Nishiki Matsubayashi, and Takushi Takata; Writing, Naonori Hu; Supervision, Hiroki Tanaka, Keiji Nihei, and Koji Ono. All authors have read and agreed to the published version of the manuscript.

## CONFLICT OF INTEREST STATEMENT

The authors declare the following financial interests/personal relationships which may be considered as potential competing interests: Naonori Hu and Hiroki Tanaka partially received research funding from Sumitomo Heavy Industries, Ltd. Taiki Nakamura, Ryusuke Kataura, Keita Suga, and Tetsuya Mukawa are employees of Sumitomo Heavy Industries, Ltd. Other authors have no conflicts of interest associated with this manuscript.
